# Lupus-Associated Autoimmune Hypophysitis: A Rare Case

**DOI:** 10.7759/cureus.92222

**Published:** 2025-09-13

**Authors:** Zhalka Abdellatif, Rami Jabareen, Nizar Hijazi

**Affiliations:** 1 Endocrinology/Internal Medicine, Emek Medical Center, Afula, ISR; 2 Internal Medicine, Emek Medical Center, Afula, ISR; 3 Rheumatology/Internal Medicine, Emek Medical Center, Afula, ISR

**Keywords:** autoimmune hypophysitis, central diabetes insipidus (cdi), male hypogonadism, pituitary gland abnormalities, systemic lupus erythematosus disease

## Abstract

Autoimmune hypophysitis (AH) is a rare inflammatory disorder of the pituitary gland, and its association with systemic lupus erythematosus (SLE) is extremely uncommon. We describe a 36-year-old man with newly diagnosed SLE who later developed fatigue, infertility, polyuria, and polydipsia. Hormonal evaluation revealed hypogonadotropic hypogonadism and low IGF-1. MRI showed pituitary stalk thickening and loss of the posterior pituitary bright spot, consistent with AH. Central diabetes insipidus (CDI) was confirmed by urine testing. The patient improved clinically after initiation of desmopressin and human chorionic gonadotropin (hCG) therapy. This case is notable because infertility and CDI were the initial manifestations of SLE-associated AH, representing an unusual clinical presentation that clinicians should be aware of.

## Introduction

Autoimmune hypophysitis (AH) is a rare inflammatory disorder characterized by lymphocytic infiltration of the pituitary gland, often resulting in varying degrees of hypopituitarism and mass effect symptoms [1]. Its presentation is frequently subtle, with nonspecific symptoms and imaging findings that may mimic sellar tumors, complicating timely diagnosis. AH most commonly affects women during late pregnancy or the postpartum period but can also occur in association with other autoimmune diseases or as an immune-related adverse event (irAE) from immune checkpoint inhibitors (ICIs), which may induce pituitary inflammation through dysregulated immune activation [2,3]. Systemic lupus erythematosus (SLE) is a multisystem autoimmune disease with heterogeneous clinical features, but hypothalamic-pituitary axis involvement is exceedingly rare and underreported [3]. The coexistence of AH and SLE is particularly uncommon, with only a few cases described in the literature [4]. Here, we report a rare case of AH in a 36-year-old man with established SLE, presenting with central diabetes insipidus (CDI). This case underscores the diagnostic challenges, therapeutic considerations, and the importance of maintaining clinical vigilance for atypical endocrine manifestations in autoimmune diseases.

## Case presentation

A 36-year-old man with a history of smoking and obesity (BMI: 36) presented with a four-month history of polyarthralgia and morning stiffness, partially responsive to NSAIDs, along with recurrent painless oral ulcers. On examination, he had mild, non-deforming synovitis involving the small joints of the hands and wrists. A solitary, painless ulcer was observed on the hard palate. There was no malar rash, discoid rash, or alopecia. Vital signs were within normal limits, and cardiorespiratory, abdominal, and neurological examinations were unremarkable. Laboratory tests revealed normocytic anemia (Hb: 12.4 g/dL), with normal renal function and electrolytes (Table 1).

**Table 1:** Complete blood count and basic metabolic panel WBC: white blood cell, RBC: red blood cell, MCV: mean corpuscular volume, MCH: mean corpuscular hemoglobin, MCHC: mean corpuscular hemoglobin concentration, RDW: red cell distribution width, BUN: blood urea nitrogen, ALP: alkaline phosphatase, AST: aspartate transaminase, ALT: alanine transaminase.

Test	Result	Normal range
WBC	7	4.0–10.0 × 10⁹/L
RBC	4.7	4.5–5.9 × 10¹²/L
Hemoglobin	12.4	13.5–17.5 g/dL
Hematocrit	37.7	41–53%
MCV	90	80–100 fL
MCH	30	27–33 pg
MCHC	33	32–36 g/dL
RDW	13.5	11.5–14.5%
Platelets	250	150–400 × 10⁹/L
Neutrophils	60	40–70%
Lymphocytes	30	20–45%
Monocytes	6	2–8%
Eosinophils	3	1–4%
Basophils	1	0–1%
Sodium	136	135–145 mmol/L
Potassium	4.2	3.5–5.0 mmol/L
Chloride	100	98–106 mmol/L
Bicarbonate (CO₂)	24	22–29 mmol/L
BUN	14	7–20 mg/dL
Creatinine	0.6	0.7–1.3 mg/dL
Glucose	129	70–99 mg/dL
Calcium	9.5	8.5–10.2 mg/dL
ALP	136	44–121 U/L
AST	38	10–40 U/L
ALT	50	7–56 U/L
Total protein	8	6.6–8.7 g/dL
Albumin	4.3	3.4–4.8 g/dL

A rheumatology consultation was obtained, and given the presence of symmetrical polyarthritis and mucosal ulcers, an autoimmune work-up was performed (Table 2). Serologic testing revealed positive antinuclear antibodies (ANA) at a titer of 1:160 in a homogeneous pattern, elevated anti-double-stranded DNA antibody levels (290 IU/mL), and low complement levels, consistent with active disease. These findings supported a diagnosis of SLE. The patient was started on prednisone (40 mg daily with taper) and hydroxychloroquine (400 mg/day). Partial clinical improvement was noted, with resolution of arthralgias and normalization of inflammatory markers.

**Table 2:** Autoimmunologic work-up

Test	Result	Normal range
Smooth muscle antibody	Negative	–
Mitochondrial antibody	Negative	–
Antinuclear antibodies (ANA)	Positive, 1:160 titer	–
Anti-dsDNA Ab	290	0.0–0.5 IU/mL
ANA pattern	Homogeneous	–
RNP	Negative	–
Anti-Smith antibody	Negative	–
SSA (Ro)	Negative	–
SSB (La)	Negative	–
SmRNP	Negative	–
Anti-CCP	Negative	<20 U/mL
Scl-70 antibody	Negative	–
Anti-Jo-1 antibody	Negative	–
Rheumatoid factor	Negative	<15 IU/mL
Complement C3	85	90–180 mg/dL
Complement C4	10	10–40 mg/dL

Approximately two months after the diagnosis of SLE, the patient was referred to the endocrinology clinic for evaluation of long-standing infertility, which had been present for several years but had not been previously investigated. Around the same time, he developed a new three-week history of polyuria and polydipsia, raising concern for an additional endocrine disorder. Endocrine evaluation (Table 3) revealed hypogonadotropic hypogonadism, with low levels of follicle-stimulating hormone (FSH), luteinizing hormone (LH), testosterone, and free androgen index, while thyroid function and prolactin levels were normal.

**Table 3:** Endocrine laboratory results

Test	Result	Normal range
Estradiol	17.3	Male: <52 pg/mL
Luteinizing hormone (LH)	1	Male: 1.5–9.3 IU/L
Follicle-stimulating hormone (FSH)	0.8	Male: 1.5–12.4 IU/L
Prolactin	7.57	Male: 2–18 ng/mL
Cortisol	16.44	Morning: 6.2–19.4 µg/dL
Thyroid-stimulating hormone (TSH)	1.46	0.4–4.0 mIU/L
Free T3	4.8	2.0–4.4 pg/mL
Free T4	12.5	10–23 pmol/L
Testosterone	0.35	Male: 1.97–6.69 ng/mL
Sex hormone-binding globulin (SHBG)	28.5	10–57 nmol/L
Free androgen index	4.7	Male: 30–150
IGF-1	57	69–211 ng/mL

**Figure 1:**
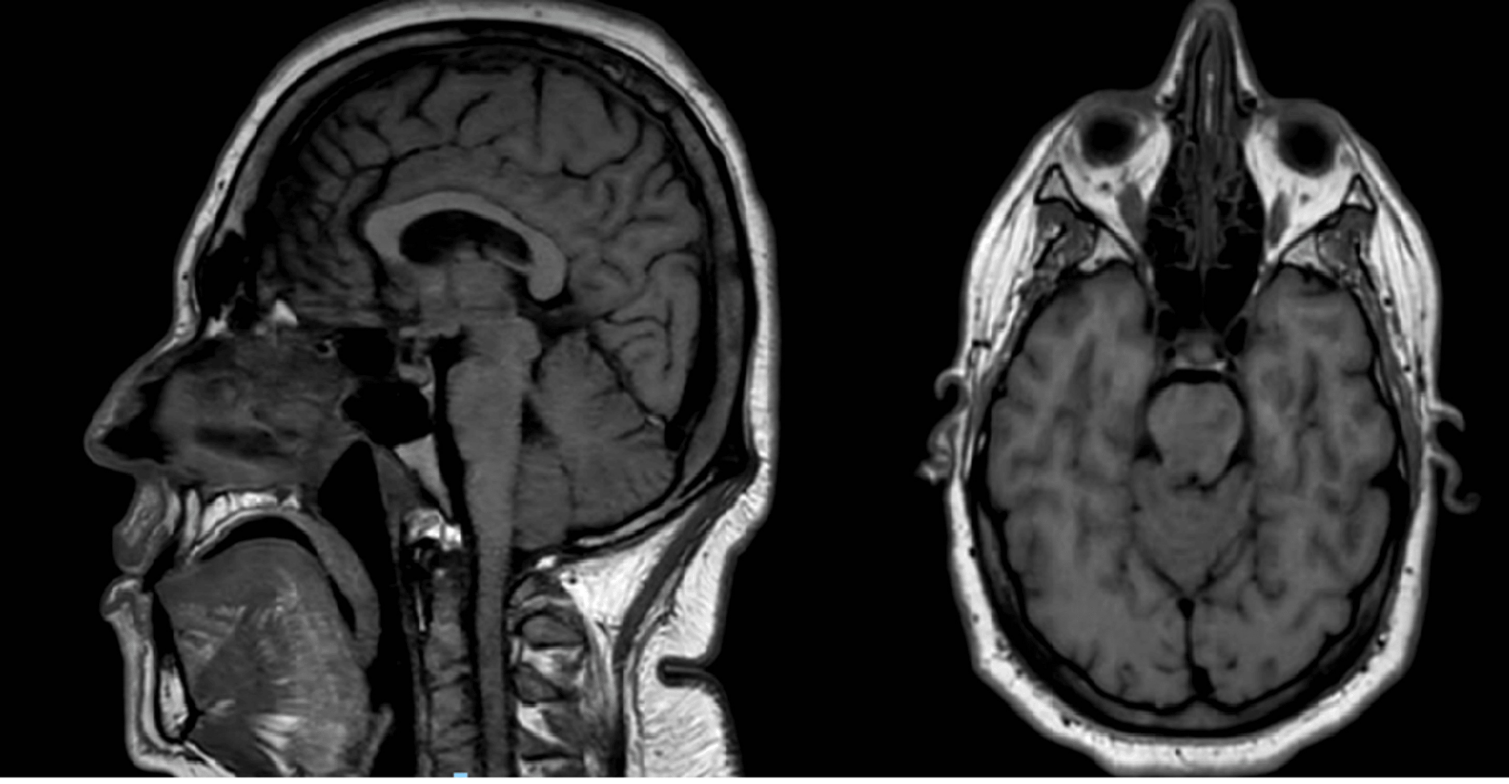
Brain MRI demonstrating pituitary stalk thickening up to approximately 4 mm, preservation of the optic chiasm, and absence of the normal posterior pituitary bright spot, suggestive of hypophysitis

A subsequent brain MRI (T1-weighted post-contrast imaging) (Figure 1) showed pituitary stalk enlargement and absence of the posterior pituitary bright spot, findings suggestive of lymphocytic hypophysitis. Given the combination of polyuria, polydipsia, and MRI abnormalities, central diabetes insipidus (CDI) was suspected. Persistent polyuria, polydipsia, and low urine osmolality in the setting of normal serum glucose supported this diagnosis. Although a formal water deprivation test was not performed due to patient discomfort, a 24-hour urine collection demonstrated hypotonic polyuria, and a marked clinical and biochemical response to desmopressin confirmed CDI. Pituitary biopsy was not pursued, as the clinical features, endocrine profile, and imaging findings were characteristic of autoimmune hypophysitis. In such typical presentations, biopsy is generally unnecessary because it is invasive and carries risks that outweigh its diagnostic value.

Desmopressin therapy was initiated for CDI, and human chorionic gonadotropin (hCG) was started to address fertility concerns.

A multidisciplinary consultation involving endocrinology, internal medicine, and rheumatology concluded that prednisone and hydroxychloroquine should be continued, with azathioprine planned as a steroid-sparing agent in the coming months.

At two-month follow-up, the patient showed clinical improvement, with resolution of polyuria and stabilization of joint symptoms.

## Discussion

AH is a rare but clinically significant condition, particularly when associated with systemic autoimmune diseases. First described in 1962 [5], AH is characterized by a disruption of the normal pituitary architecture due to lymphocytic infiltration, often resulting in glandular dysfunction. Clinically, it typically presents as a sellar mass, with symptoms such as headache and visual disturbances arising from pituitary enlargement caused by inflammation and edema [5]. The disease course is variable, ranging from spontaneous or treatment-induced remission to progressive destruction of the pituitary tissue, ultimately leading to fibrosis and permanent hormonal deficiencies. This dysfunction may involve reduced secretion of ACTH, TSH, gonadotropins, or prolactin [5].

Although definitive diagnosis traditionally requires histopathological confirmation via pituitary biopsy, cases with established glandular atrophy can often be diagnosed non-invasively using clinical evaluation and imaging findings [6]. While tissue confirmation was not pursued in our patient due to its invasiveness and limited impact on management, this remains a recognized limitation in the diagnostic approach.

SLE is a chronic autoimmune disorder affecting multiple organ systems, including the skin, kidneys, lungs, central nervous system, and heart [7]. Although both SLE and AH are autoimmune in nature, the pathophysiological link between them remains unclear. To date, there is no histologic evidence definitively identifying the pituitary gland as a direct target of SLE. However, emerging case reports [3,8-11], including ours, suggest a possible association.

To better understand the clinical spectrum of AH in SLE, we reviewed previously reported cases. Five cases of AH associated with SLE [3,8-11] show a predominance in young adults, with four patients aged 19-27 and one older male aged 66. Three patients were female, and two were male. Most initially presented with SLE-related symptoms and later developed features suggestive of hypophysitis, including visual disturbances, headache, nausea, polyuria, and polydipsia. One patient had a prior diagnosis of diabetes insipidus and subsequently developed systemic symptoms such as facial erythema and malaise.

Diagnosis in these cases was primarily based on clinical features, laboratory findings, and imaging studies. Surgical intervention confirmed the diagnosis histologically in two cases [3,9]. All five patients received prednisone and hydroxychloroquine as part of their treatment. Three achieved remission, one patient died due to severe hyponatremia [11], and the fifth experienced long-term resolution of hypopituitarism and hyponatremia after treatment with glucocorticoids and immunosuppressive therapy [8].

Azathioprine was chosen in our case due to its established role as a steroid-sparing immunosuppressant in autoimmune conditions, including AH, particularly in the context of SLE where evidence for other agents is limited. Compared with cyclophosphamide, azathioprine has a more favorable side-effect profile for long-term use, and it is preferred over mycophenolate when lymphocytic infiltration is suspected rather than organ-threatening disease. Although data are scarce, case reports and small series have shown clinical improvement in SLE-related AH with azathioprine, supporting its selection in this patient. This approach allows gradual reduction of corticosteroid therapy while targeting the underlying autoimmune activity [12,13]. 

Our case is distinguished by the patient’s presentation with infertility in combination with features of CDI, a combination not emphasized in previous reports. While polyuria and polydipsia have been noted previously, the presence of long-standing infertility, together with laboratory-confirmed hypogonadotropic hypogonadism and CDI, highlights an important clinical clue often overlooked. This case underscores the importance of considering AH in SLE patients presenting not only with common symptoms such as headache or mass effect but also with atypical features such as infertility or signs suggestive of CDI. Early recognition of these manifestations may facilitate timely diagnosis and appropriate treatment, potentially reducing long-term endocrine morbidity.

## Conclusions

This case highlights the importance of considering autoimmune hypophysitis in patients with SLE who develop endocrine symptoms such as polyuria, polydipsia, or reproductive dysfunction. Pituitary involvement should be suspected even in the absence of classic features, as early recognition may help prevent irreversible hormone deficiency. It also underscores the gaps in understanding the mechanisms and evidence-based management of SLE-related hypophysitis, emphasizing the need for well-designed studies to guide diagnosis and treatment.
